# RNA-Seq Analysis and *De Novo* Transcriptome Assembly of Jerusalem Artichoke (*Helianthus tuberosus* Linne)

**DOI:** 10.1371/journal.pone.0111982

**Published:** 2014-11-06

**Authors:** Won Yong Jung, Sang Sook Lee, Chul Wook Kim, Hyun-Soon Kim, Sung Ran Min, Jae Sun Moon, Suk-Yoon Kwon, Jae-Heung Jeon, Hye Sun Cho

**Affiliations:** 1 Plant Systems Engineering Research Center, Korea Research Institute of Bioscience and Biotechnology, Daejeon, Korea; 2 Animal Material Engineering, Gyeongnam National University of Science and Technology, Jinju, Korea; The Chinese University of Hong Kong, Hong Kong

## Abstract

Jerusalem artichoke (*Helianthus tuberosus* L.) has long been cultivated as a vegetable and as a source of fructans (inulin) for pharmaceutical applications in diabetes and obesity prevention. However, transcriptomic and genomic data for Jerusalem artichoke remain scarce. In this study, Illumina RNA sequencing (RNA-Seq) was performed on samples from Jerusalem artichoke leaves, roots, stems and two different tuber tissues (early and late tuber development). Data were used for *de* *novo* assembly and characterization of the transcriptome. In total 206,215,632 paired-end reads were generated. These were assembled into 66,322 loci with 272,548 transcripts. Loci were annotated by querying against the NCBI non-redundant, Phytozome and UniProt databases, and 40,215 loci were homologous to existing database sequences. Gene Ontology terms were assigned to 19,848 loci, 15,434 loci were matched to 25 Clusters of Eukaryotic Orthologous Groups classifications, and 11,844 loci were classified into 142 Kyoto Encyclopedia of Genes and Genomes pathways. The assembled loci also contained 10,778 potential simple sequence repeats. The newly assembled transcriptome was used to identify loci with tissue-specific differential expression patterns. In total, 670 loci exhibited tissue-specific expression, and a subset of these were confirmed using RT-PCR and qRT-PCR. Gene expression related to inulin biosynthesis in tuber tissue was also investigated. Exsiting genetic and genomic data for *H. tuberosus* are scarce. The sequence resources developed in this study will enable the analysis of thousands of transcripts and will thus accelerate marker-assisted breeding studies and studies of inulin biosynthesis in Jerusalem artichoke.

## Introduction

The sunflower species Jerusalem artichoke (*Helianthus tuberosus* L.), in the family Asteraceae of the order Asterales, has been cultivated as a vegetable, a fodder crop, and a source of inulin for food and industrial purposes [1]–[4]. Jerusalem artichoke, which has been cultivated since the 17^th^ century, can grow well in nutritionally poor soil and has good resistance to frost and plant diseases [5], . In the early 1900s, systematic breeding programs began to explore the use of *H. tuberosus* tubers for industrial applications such as the production of ethanol [4]. Jerusalem artichoke is a hexaploid with 102 chromosomes (2n = 6× = 102) [7] that is thought to have originated in the north-central U.S., although the exact origins remain a subject of debate [8], [9]. Despite its cultural and economic significance, few studies have investigated the genetic origins of Jerusalem artichoke and its various cultivars. A recent study assessed the origin of Jerusalem artichoke using genome skimming [10], a new technique for assembling and analyzing the complete plastome, partial mitochondrial genome, and nuclear ribosomal DNA genomes. This analysis showed that the genome of Jerusalem artichoke was not derived from *Helianthus annuus* (an annual) but instead originated from perennial sunflowers through hybridization of the tetraploid Hairy Sunflower (*Helianthus hirsutus*) with the diploid Sawtooth Sunflower (*Helianthus grosseserratus*). [11], [12]. These results indicate that *H. tuberosus* is an alloploid species, having a set of chromosomes from each progenitor and double the chromosome number of the two parental species.

Many members of the Asteraceae family accumulate fructans (fructose polymers) in underground storage organs [13]. On such fructan is, inulin, which is stored in the vacuole in approximately 15% of flowering plant species [14]. Jerusalem artichoke and chicory (*Cichorium intybus* L.) are the most important cultivated sources of inulin [15]–[17]. Inulin molecules are much smaller than starch molecules, and have 2−70 linked fructose moieties terminated by a glucose residue [7]. The average number of fructose subunits depends on the species, production conditions, and developmental timing [18]. Inulin has many uses in the production of food [19], [20], and pharmaceuticals [21]–[23], and can be used as a storage carbohydrate for bioethanol production [24]. The inulin produced by Jerusalem artichoke is therefore a commercially valuable resource [7].

Recent advances in next-generation sequencing technology have enabled gene discovery, analysis of gene content, and measurement of gene expression in non-model organisms that lack a published genome sequence. For example, transcriptome sequencing can be used for genome-wide determination of absolute transcript levels, identification of transcripts, and delineation of transcript structure (including 5′ and 3′ ends, introns, and exons) [25]–[28]. Transcriptome sequencing can also identify genetic variations such as, single nucleotide polymorphisms (SNPs) and simple sequence repeats (SSRs) [29]. In recent years, RNA-Seq analysis has facilitated transcriptome characterization in hundreds of plant species lacking sequenced genomes [30]–[34].

In this study, we used RNA-Seq technology to develop the first *H. tuberosus* transcriptome dataset. *De novo* transcriptome sequencing was performed on RNA from five different *H tuberosus* tissues. We identified 66,322 loci, annotated 40,215 loci, and mapped 11,844 loci to 237 Kyoto Encyclopedia of Genes and Genomes (KEGG) pathways. We also identified 670 tissue-specific candidate loci and 10,778 SSRs. This novel dataset will be an important resource in the further genetic characterization of Jerusalem artichoke and will be particularly valuable in marker-assisted breeding and investigation of traits related to inulin biosynthesis.

## Materials and Methods

### 2.1 Plant Materials and RNA Isolation

A widely-cultivated Jerusalem artichoke cultivar, Purple Jerusalem Artichoke (PJA), was used for transcriptome analysis. PJA tubers were planted in January 2012 and were grown under normal conditions until harvesting. Stems, leaves, and tubers (stages 1 and 2; tuber1 and tuber2, respectively) were collected 6 months after planting. To avoid contamination with pathogen, roots were collected from *in* *vitro*-cultivated PJA. Tissues were snap-frozen in nitrogen upon harvest and were stored at −80°C until further processing. Total RNAs were extracted using Trizol Reagent (Invitrogen, Carlsbad, CA, USA), and were then treated with DNase I (Fermentas, Pittsburgh, PA, USA) according to the manufacturers’ instructions. The OD260/230 ratio was determined using a NanoDrop ND-1000 Spectrophotometer (Thermo Fisher Scientific, Wilmington, DE, USA) and was used for assessment of RNA quality and purity.

### 2.2 Transcriptome Sequencing

An equal amount of total RNA from each tissue was pooled for transcriptome sequencing in order to obtain a comprehensive range of transcripts. Poly(A)+ RNAs were purified from the pooled total RNA (20 µg) using oligo(dT) Dynabeads. Impurities were removed from the hybridized sample using a series of low-salt washes. First-strand cDNAs were synthesized using oligo(dT) primers. RNA was then degraded with RNase H (Invitrogen, Carlsbad, CA, USA) and second-strand cDNA were synthesized using DNA polymerase I (New England BioLabs, Ipswich, MA, USA). Double-stranded cDNAs were randomly fragmented using a nebulizer. The fragments were then repaired and extended at the 3′ end by addition of a single adenine, and different adapters were ligated to the 5′ and 3′ ends. The ligated fragments were separated on a gel, and fragments of ∼200 bp were isolated. After amplification by polymerase chain reaction (PCR), fragments were separated using electrophoresis, purified, and subjected to Illumina HiSeq2000 sequencing. Raw sequence data were generated by the Illumina analysis pipeline. Sequence data are deposited in the NCBI Sequence Read Archive (SRA, http://www.ncbi.nlm.nih.gov/Traces/sra) under study number PRJNA258432.

### 2.3 *De novo* Transcriptome Assembly

Raw sequence data were filtered using standard RNA-Seq parameters. Briefly, low-quality and N-base reads were trimmed from the raw reads and reads were filtered by Phred quality score (Q≥20 for all bases) and read length (≥25 bp). The 3′ ends of the clean reads were trimmed to form five sets of reads from the five different tissues. These datasets were then pooled and assembled using *de* *novo* assemblers (Velvet v1.2.07 [35] and Oases v0.2.08 [36]) based on the de Bruijn graph algorithm. Reads were assembled into contigs at distinct k-mer values (45, 51, 53, 55, 57, 59, 61, 63, 65, 67, 69 and 75) using Velvet. Contigs at each k-mer value were assembled into transcripts using Oases. Finally, the transcripts assembled at k-mer values 63 and 65 were merged using Oases with a minimum length of 200 bp and other default settings. Hash length (k-mer = 65) was considered for selection of the optimal *de* *novo* assembly as described previously [37]. The cleaned reads were also assembled using Trinity release_2011-11-26 [38] with k-mer of 25, minimum k-mer coverage of 1. Default settings were used for all other parameters. The performance of the two assembly tools was assessed at N50 value, mean length, maximum length and transcript number. Data sets produced using Velvet-Oases were selected for subsequent analyses. Singletons and the longest sequence in each cluster were designated as loci and were then translated in all six frames. Putative transcripts were validated by comparison with gene sequences in the Phytozyme database (http://www.phytozyme.net/) using BLASTX (E-value ≤1E-05, BLAST v.2.2.28+). In addition, the assembled loci were compared with expressed sequence tag (EST) sequences from *H. tuberosus* (a total of 40,388 ESTs) and *H. annus* (a total of 134,474 ESTs) in NCBI GenBank (ftp://ftp.ncbi.nih.gov/pub/TraceDB/helianthus_tuberosus/ and http://www.ncbi.nlm.nih.gov/Taxonomy/Browser/wwwtax.cgi?id=4232, respectively) using BLASTN [39] with an E-value cut-off of 1E-20.

### 2.4 Functional Annotation and Classification

BLASTX and Blast2GO software v2.4.4 [40] were used to compare the assembled loci (≥200 bp) to the NR, Phytozome, and UniProt databases at a threshold E-value ≤1.0E-05. For Gene Ontology analysis, the gene ontology (GO) database (http://www.geneontology.org/) was downloaded and the assembled loci were annotated to the GO database using BLASTP (E-value ≤1.0E-06). GO term annotation was determined using GO classification results from the Map2Slim.pl script [37]. Protein sequences with the highest sequence similarities and cut-offs were retrieved for analysis. Further functional enrichment analysis was carried out using DAVID [41], [42] and AgriGO (plant GO slim, FDR≤0.01) [43]. Gene lists were annotated by TAIR ID, and were analyzed with default criteria (counts ≥2 and EASE score ≤0.1) for GO terms [44], Clusters of Eukaryotic Orthologous Groups (KOGs) [45], and KEGG pathways [46]. In addition, KEGG pathways were assigned to the locus sequences using the single-directional best hit method on the KEGG Automatic Annotation Server [47], [48].

Coding sequences were predicted through BLAST comparisons with public protein databases. Sequences were compared with the Phytozome and Nr protein databases using BLASTX (E-value ≤1.0E-5). Loci that matched sequences in the Phytozome database were not examined further. Coding sequences were derived from loci sequences according to BLASTX outcomes (≥200 bp). In addition, full-length transcripts were predicted using BLASTP with the following parameters to ensure similarity of transcripts: orthologous gene of 99% similarity, minimum 90% identity.

### 2.5 Analysis of Differential Expression and Tissue-Specific Loci

Five mRNA libraries were generated from separate tissues using Illumina sequencing. Reads for each sequenced tag were mapped to the assembled loci using Bowtie (mismatch ≤2 bp, other parameters as default), and the number of clean mapped reads for each locus was counted. The DEGseq package [49] was used to identify differentially expressed genes. The five different libraries were compared pairwise using a greater than two-fold difference as the criterion for differential expression. Significant differential expression between tissues was defined by *p*-value < 0.001, FDR < 0.01, and log_2_ > 2. Differential expression analysis between tissues was used to identify candidate loci with tissue-specific expressions, and to determine functionally enriched loci, as described above.

Tissue-specific loci were selected based on the read counts from leaf, root, stem, tuber1 and tuber2 samples of *H. tuberosus*. Tissue-specific candidates were those with > 200 reads from the target tissue and < 50 reads from other tissues.

### 2.6 Identification of SSRs

SSRs were detected using the MIcroSAtellite Identification Tool (MISA, http://pgrc.inpk-gatersleben.de/misa/) Perl script. The assembled unigene sequences were screened for mono-, di-, tri-, tetra-, penta- and hexa-nucleotide repeat motifs with a minimum repeat number of 10, 6, 5, 5, 5 and 5, respectively. A maximum distance of 100 nucleotides was allowed between two SSRs.

### 2.7 Reverse Transcription (RT) and Quantitative Reverse Transcription (qRT) PCR Analyses

Total RNA was isolated from five *H. tuberosus* tissues using RNAiso Plus (Takara, Tokyo, Japan). The cDNAs were synthesized with M-MLV reverse transcriptase and an oligo(dT) primer in a 20 µL volume according to the manufacturer’s instructions (Invitrogen, Carlsbad, CA, USA). Twenty putative tissue-specific genes (five per tissue type), were selected for RT-PCR. Quantitative RT-PCR was performed in 10 µL reactions containing gene-specific primers, 1 µL cDNA as template, and SYBR Premix Ex Taq. Reactions were performed using a CFX96 Real-Time PCR system (BioRad, Hercules, CA, USA). The thermal profile for qRT-PCR was as follows: 3 min at 95°C, followed by 40 cycles each consisting of 95°C for 25 sec, 60°C for 25 sec and 72°C for 25 sec. Primer specificities and the formation of primer-dimers were monitored by dissociation curve analysis. The expression level of *H. tuberosus Actin2* (*HtActin2*) was used as an internal standard for normalization of cDNA template quantity. RT-PCR and qRT-PCR reactions were performed in triplicate.

## Results and Discussion

### 3.1 RNA-sequencing and *de* *novo* Transcriptome Assembly of *H. tuberosus*

Total RNAs were isolated from five different tissues of the PJA cultivar: leaves, stems, roots, tuberous initial stage 1 (tuber1) and mature stage 2 (tuber2). The extracted RNAs were then mixed in equal proportions for mRNA isolation, fragmentation, cDNA synthesis, and sequencing. RNA sequencing with the Illumina Hiseq2000 produced 244,101,906 paired-end 101 bp reads corresponding to more than 24.4 billion base pairs of sequence. The raw reads were subjected to quality control using FastQC, and reads were trimmed (Table S1). The total number of high-quality reads was 206,215,632, and these contained a total of 16,675,072,220 nucleotides. Of these, 68.37% reached a strict quality score threshold of Q ≥20 bases and read length ≥25 bp, and these were used for *de* *novo* assembly [31].

The clean RAN-Seq reads were assembled *de* *novo* into contigs using two assemblers with optimal parameters. First, the reads were assembled using Velvet-Oases (k-mer = 65) [35], [36] to reduce redundancy and generate longer sequences: 66,322 loci and 272,548 transcripts with lengths ≥200 bp were produced. Second, the reads were assembled using the Trinity program [38]: 246,155 transcripts with lengths ≥200 bp were produced. A comparison of transcript length distribution between the two assemblies is shown in Figure S1. Overall, the mean length, maximum length, and N50 were longer for the Velvet-Oases assembled sequences than for the Trinity assembled sequences and we therefore used the Velvet-Oases assembly for subsequent analyses.

The sequences assembled by Velvet-Oases were ≥200 bp and had an average length of 761 bp (a total of 4,083,193,637 bp), N50 length of 1,249 bp, and maximal length of 15,368 bp. Transcript sequences were also ≥200 bp and had an average length of 1,176 bp (a total of 16,675,072,220 bp), N50 length of 1,703 bp, and maximal length of 16,437 bp (Table 1). A substantial number of transcripts (124,741) had lengths > 1 kb. These transcripts were clustered, resulting in 66,322 loci that included 16,013 loci (24.1%) > 1 kb in length (Table 1). The assembled sequences are deposited at http://112.220.192.2/htu and are summarized in Table S2. In summary, we generated genome-wide locus sequences of *H. tuberosus*, a resource that will promote functional genomics approaches in Jerusalem artichoke.

**Table 1:** Summary of *H. tuberosus de* *novo* assembly using Velvet-Oases.

		Locus	Transcripts
**Number of sequences**		66,322	272,548
**Sequence statistics**	Minimum	200	200
	Maximum	15,368	16,437
	mean length	761	1,176
	N50	1,249	1,703
**Distribution of sequence lengths**	≤500 bp	36,383	79,718
	501≤1,000 bp	13,926	68,089
	1,001≤1,500 bp	7,027	47,719
	1,501≤2,000 bp	4,216	33,486
	2,001≤	4,770	43,536

### 3.2 Validation of Assembled Loci Against Publically Available ESTs from *H. tuberosus*

We used publically available EST data to validate the loci identified by our RNA-Seq and assembly. Sequence information for ESTs from *H. tuberosus* was retrived from the NCBI GenBank database (most recently accessed in January, 2014). BLASTN analysis of the assembled loci was performed against the *H. tuberosus* ESTs (40,388 ESTs) and the best hit for each locus was selected. Of the *H. tuberosus* ESTs, 35,402 sequences (87.65%) matched a locus from our assembly, but no match was found for 4,986 ESTs (12.35%). Most of the loci with hit matched the ESTs with good coverage and assembly quality (Figure S2A). Of our 66,322 loci, 52,174 loci showed no BLAST hits to the *H. tuberosus* ESTs and were thus considered to be putative transcripts newly-identified by our RNA-Seq analysis.

Transcriptome information is not available for the direct progenitors of *H. tuberosus*, *Helianthus hirsutus* and *Helianthus grosseserratus*; however, a curated unigene collection for sunflower (*Helianthus annuus* L.) was recently generated by EST assembly analysis [50]. We used BLASTN to compare our assembled *H. tuberosus* loci against the ESTs of *H. annuus* and found that 81.04% of *H. annuus* ESTs (108,984 out of 134,474) had matches among the *H. tuberosus* loci (Figure S2B).

### 3.3 Functional Annotation of *H. tuberosus* Loci

**Figure 1:**
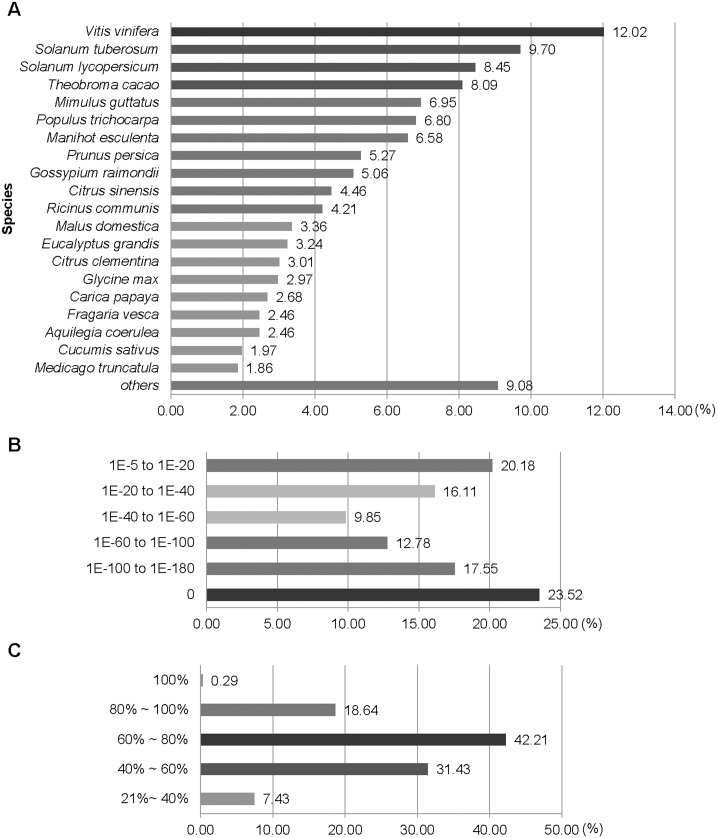
Comparison of assembled *H. tuberosus* loci with database sequences. Species, E-value, and similarity distributions of the assembled loci against database sequences are shown. (**A**) Species distribution of the top BLAST hits for the assembled loci (Cut-off, E-value = 0). (**B**) E-value distribution of BLAST hits for the assembled loci (E-value ≤1.0e-05). (**C**) Similarity distribution of BLAST hits for the assembled loci.

After filtering out short-length and low-quality sequences, we used our assembled locus sequences to perform similarity searches against public protein databases (Phytozome [51] Nr [52], and UniProt [53]). Firstly, we searched all six frame translations of our loci against the Phytozyme protein database using BLASTX (E-value ≤1.0E-05). Database matches were found for 32,746 loci (49.4%). The unmatched loci were further analyzed against the NCBI non-redundant (Nr) and UniProt database. Additionally, databases were searched using BLASTN and BLASTX to identify homologous genes. Overall, 40,215 loci (60.64%) matched significantly similar sequences within the databases. The 39.36% of sequences (26,107 loci) without hits may represent novel loci specific to *H. tuberosus*. Alternatively, these sequences may have been too short to produce significant hits. Similar search outcomes have been observed in previous non-model plant studies [54]–[56] (Table 2). Based on the top BLASTX hits against the Phytozome database, *H. tuberosus* loci were most similar to sequences from *Vitis vinifera* (3,556 loci, 12.02%) followed by *Solanum tuberosum* (2,869 loci, 9.7%) and *Solanum lycopersicum* (2,500 loci, 8.45%) (Figure 1A). The E-value distribution of the top matches showed that 23.52% of the sequences had an extremely high E-value score (E-value = 0) and 76.48% of the homologous sequences had values in the range 1.0E-05−1.0E-180 (Figure 1B). The similarity distribution showed that 18.93% of these sequences had similarities greater than 80%, 42.21% had similarities of 60%−80%, and 38.86% had similarities < 60% (Figure 1C).

**Table 2:** Summary of annotations of assembled *H. tuberosus* sequences.

Assembly	Total sequences	Phytozome	Nr	Uniprot	KOG	KEGG	TAIR	GO	Total annotations
Number oftranscripts	272,548	185,145	41,334	208,271	95,980	35,180	153,573	120,320	192,965
Number ofloci	66,322	32,746	32,156	38,668	15,434	11,844	28,781	19,848	40,215

Loci with matches in the protein databases were examined further. The translated the coding sequences of these loci had ≥90% identity with the matched sequences. Of the annotated 40,215 loci, 10,066 contained a putative full-length transcript (with 3′ and 5′ untranslated regions). BLAST analysis using those loci indicated that information from other species was sufficient to allow annotation of the *H. tuberosus* loci.

### 3.4 Classification of *H. tuberosus* Loci

**Figure 2:**
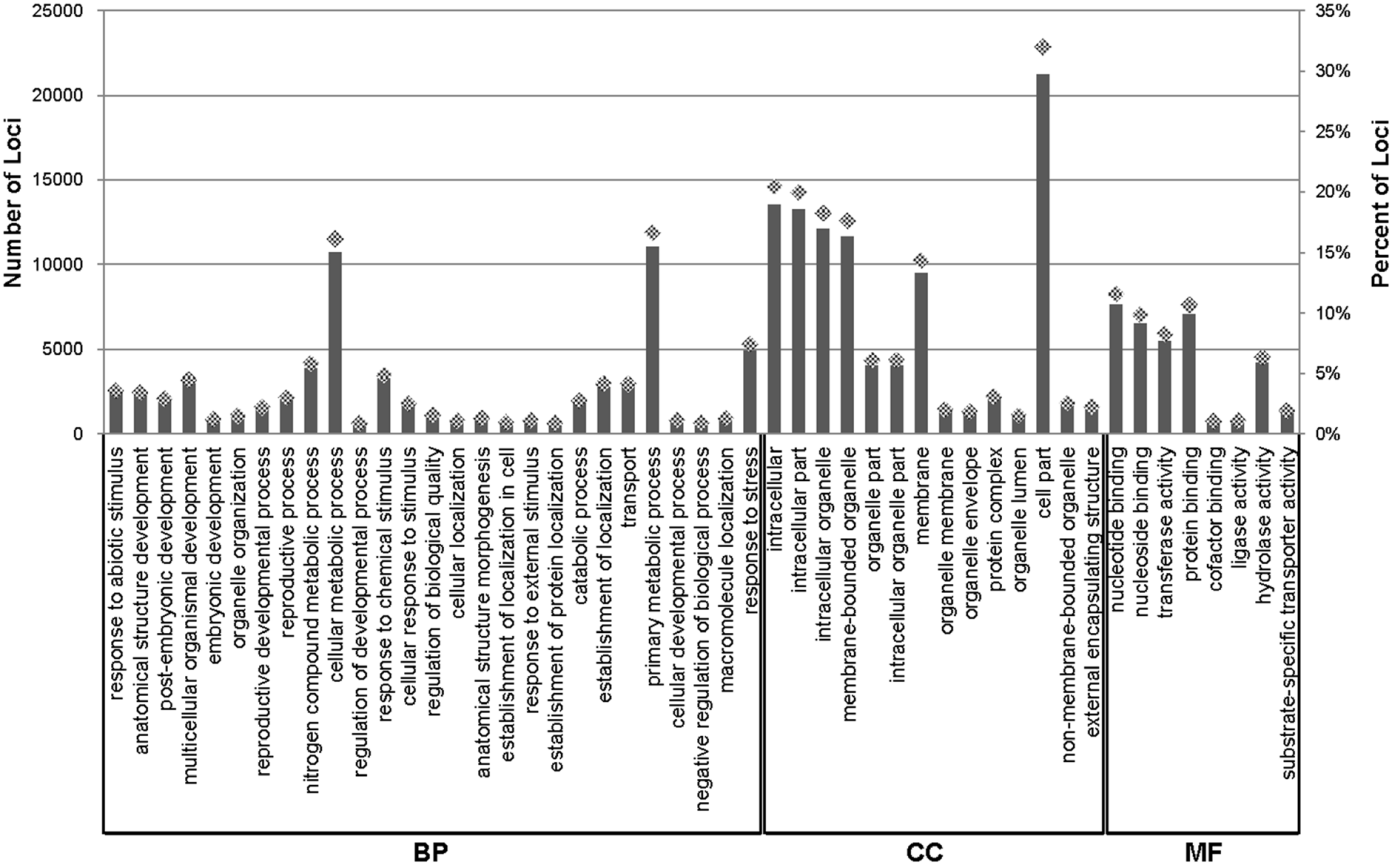
Gene Ontology (GO) classification of the assembled loci. The results of BLASTX searches against the Phytozome database were used for GO term mapping and annotation. The number and ratio of sequences assigned to level 2 GO terms from GO subcategories including biological process, molecular process, molecular function, and cellular component are shown (BP: biological process, CC: Cellular Component, MF: Molecular Function).

We used GO term enrichment analysis to classify the functions of the assembled *H. tuberosus* loci [44]. The BLASTX similarity search results for the 66,322 *H. tuberosus* loci were imported into the Phytozome database for GO mapping and annotation with TAIR information. Sequence annotations associated with 19,848 loci (29.93%) were categorized into the three main GO ontologies: biological process (BP), cellular component (CC), and molecular function (MF) (Figure 2). In total, 7,589, 8,685 and 8,510 loci were assigned GO terms from the BP, CC, and MF categories, respectively. The GO terms were summarized into 49 subcategories with GO classifications at level 2. In the BP category, the dominant subcategories assigned to *H. tuberosus* loci were as follows: ‘Primary metabolic process’ (15.19%), ‘Cellular metabolic process’ (14.75%), ‘Response to stress’ (6.76%), ‘Nitrogen compound metabolic process’ (5.33%) and ‘multicellular organismal development’ (4.08%). In the CC category, ‘Cell part’ (21.61%), ‘Intracellular’ (13.81%), ‘Intracellular part’ (13.49%), ‘Intracellular organelle’ (12.33%), and ‘Membrane-bounded organelle’ (11.89%) were the dominant subcategories. Finally, ‘Nucleotide binding’ (22.12%), ‘Protein binding’ (20.45%), ‘Nucleoside binding’ (18.94%), ‘Transferase activity’ (15.80%), and ‘Hydrolase activity’ (12.17%) were dominant in the MF category. These annotations indicated that extensive membrane metabolic activity occurred in *H. tuberous* in the sampled tissues. The loci were analyzed further for GO-category enrichment relative to Plant GO slim categories using AgriGO [43]. The *H. tuberosus* loci contained 71 significantly enriched (FDR ≤ 0.01) functional GO terms in the BP category, including top five terms (“cellular process”, GO:0009987; “cellular metabolic process”, GO:0044237; “metabolic process”, GO:0008152, “primary metabolic process”, GO:0044238, and “response to stimulus”, GO:0050896, respectively). The GO term “cellular, macromolecule, nitrogen compound and primary metabolic process” was highly enriched (FDR≤1.0E-40), and enriched daughter terms included “nucleobase, nucleoside, nucleotide and nucleic acid metabolic process” (GO:0006139), “cellular macromolecule metabolic process” (GO:0044260), “macromolecule modification” (GO:0043412), “carbohydrate metabolic process” (GO:0005975; including several loci with fructan 1,2-beta-fructan 1-fructosyltransferase, invertase, hexokinase, sucrose synthase, sucrose phosphate synthase, starch synthase, starch branching enzyme, and beta glucosidase sequences), and “cellular biosynthetic process” (GO:00044249; sucrose 1F-beta-D-fructosyltransferase). These results suggest that gene expression in *H. tuberosus* is geared towards carbohydrate metabolism, cellular biosynthetic processes, and macromolecule modification functions. This expression enrichment concurs with biosynthetic analysis results indicating that inulin accumulation occurs at the time of tuber initiation [4], [19]. An additional enriched GO term was “protein modification process” (GO:0006464). This included loci with cyclophilin, FKBP-type peptidyl-prolyl cis-trans isomerase, CONSTANS-like 4, heat shock protein 7, chaperones protein chaperone, and transferase sequences. As in the MF category, loci were associated with 16 significantly enriched GO terms. These included the level two terms “catalytic activity” (GO:0003824), “binding” (GO:0005488), “transporter activity” (GO:0005215), and “receptor activity” (GO:0004872), the level three terms “protein binding” (GO:0005515), “transferase activity” (GO:0016740), and “hydrolase activity” (GO:0016787), and the level four terms “transferase activity, transferring phosphorus-containing groups” (GO:0016772) and “hydrolase activity, acting on acid anhydrides” (GO:0016817, including several fructosyltransferase loci). The most significantly enriched of these was the level two term “catalytic activity”. In the CC category, the GO terms “cytoplasmic part” (GO:0044444), “interacellular membrane-bounded organelle” (GO:0043231), “interacellular organelle part” (GO:0044446) and their daughter terms (“plastid”, “Golgi apparatus”, “cytosol” and “vacuole”) were highly enriched (FDR≤1.0E-60). These enrichments correspond with the involvement of storage organelles in tuber inulin accumulation. The “vacuole” term was also found to be significantly enriched in tuber samples. The *H. tuberosus* annotation results were similar to those from the potato and sweet potato transcriptomes [57]–[60]. The majority of the sequenced *H. tuberosus* loci were associated with fundamental regulatory and metabolic processes in the membrane.

**Figure 3:**
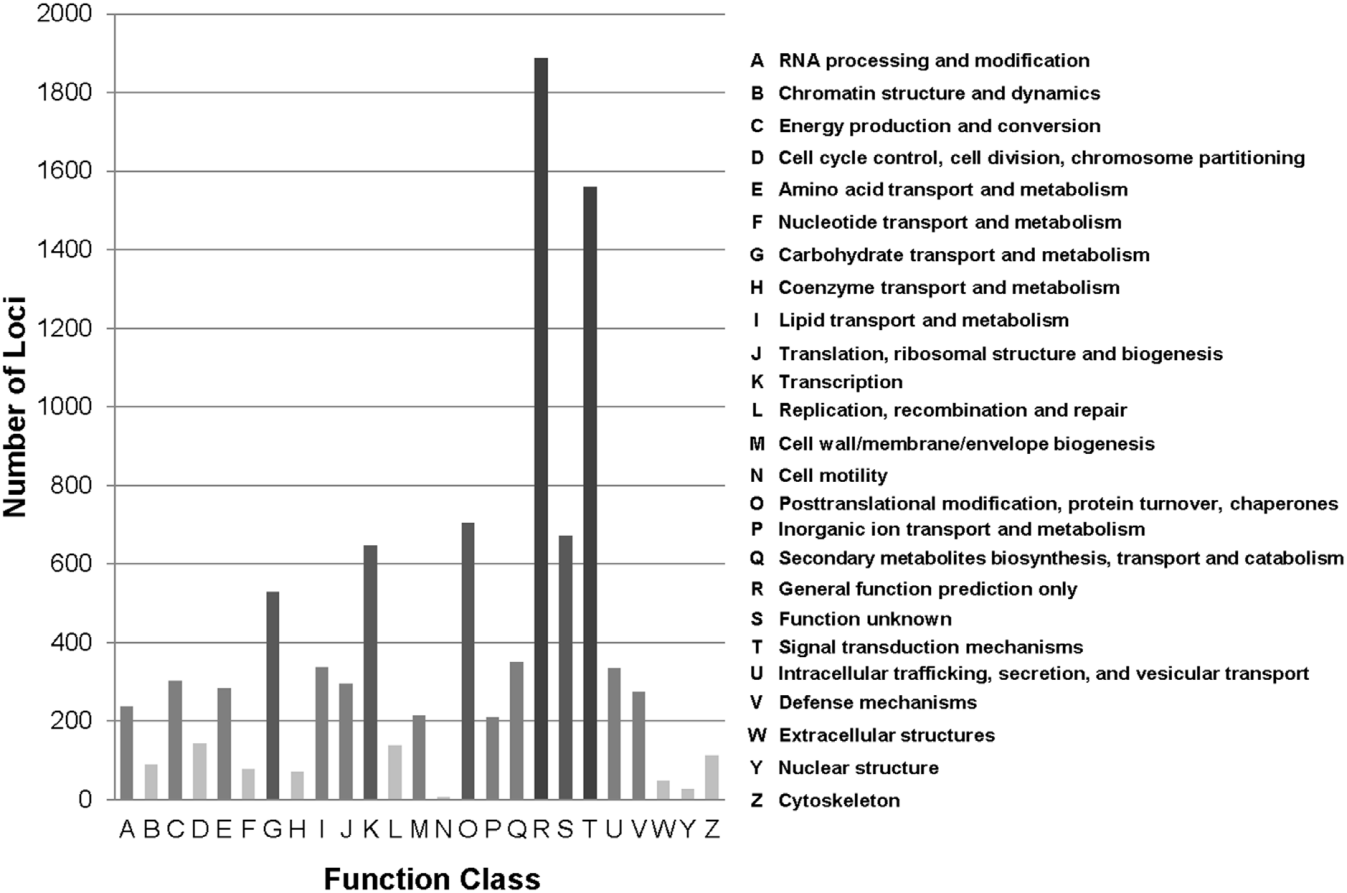
Eukaryotic Orthologous Groups (KOG) classification of the assembled loci. Of 66,322 loci with Nr, Phytozome and UniProt hits, 15,434 sequences with significant homologies in the KOG database (E-value ≤1.0E-5) were classified into 25 categories.

To assess the functionality of the *H. tuberosus* transcriptome, the annotated loci were matched to the Eukaryotic Orthologous Groups (KOGs) database to find homologous genes. The search outcomes were used to determine sequence directions within loci [45]. The 66,322 loci were annotated with 15,434 KOG terms in 25 classifications (Figure 3). Each KOG term represents a conserved domain; therefore, these results indicated that a large proportion of the putative proteins encoded by the assembled locus sequences had protein domains with existing functional annotations [45]. The cluster for ‘General function’ prediction (19.77%) was the most frequently identified group, followed by ‘Signal transduction mechanisms’ (16.34%), ‘Post translational modification, protein turnover, chaperones’ (7.37%), ‘Function unknown’ (7.03%), ‘Transcription’ (6.78%), ‘Carbohydrate transport and metabolism’ (5.53%), and ‘Secondary metabolites biosynthesis, transport and catabolism’ (3.67%).

**Figure 4:**
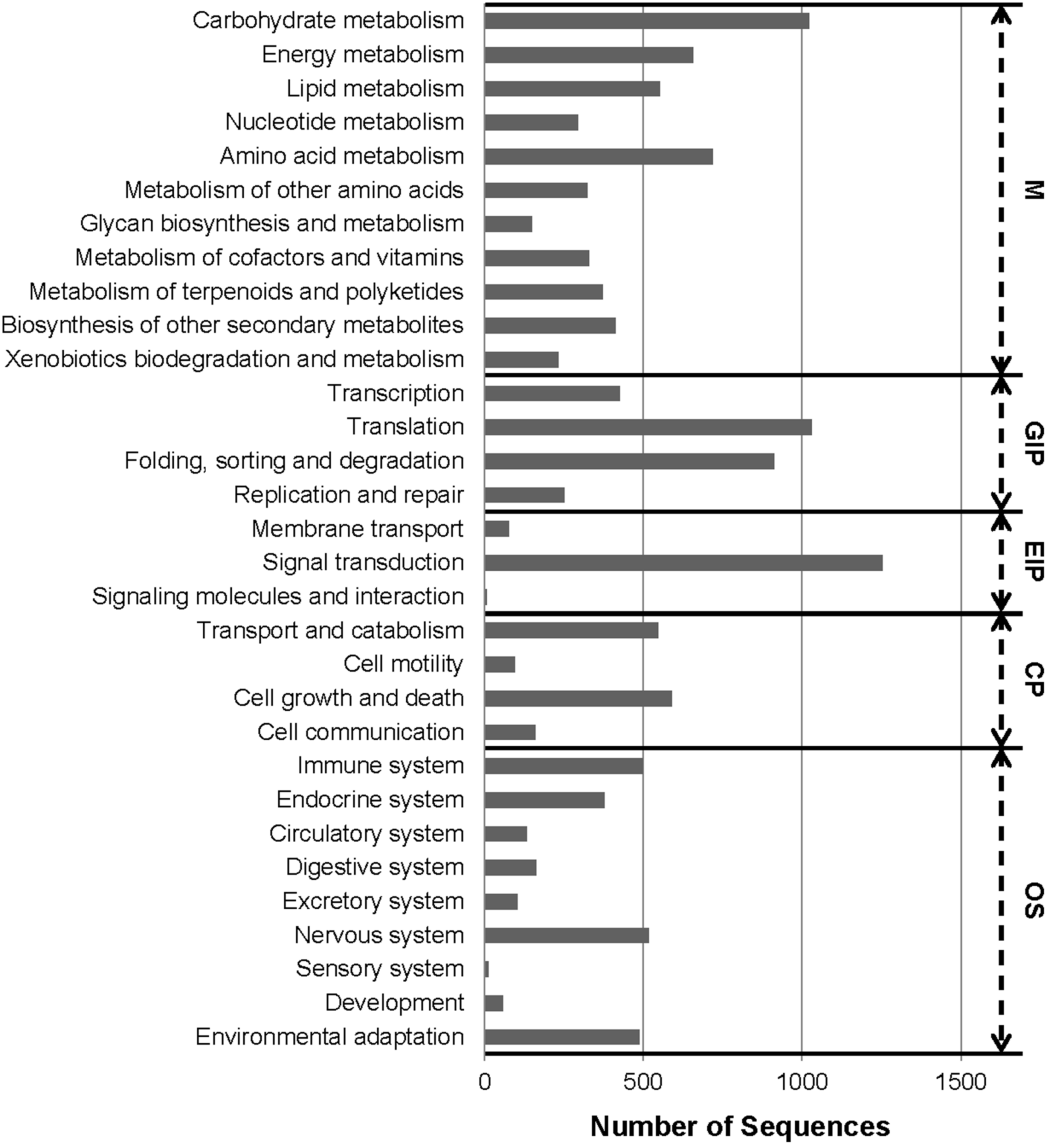
Kyoto Encyclopedia of Genes and Genomes (KEGG) classification of the assembled loci. Locus sequences were compared using BLASTX with an E-value cut-off ≤1.0E-05 against the KEGG biological pathways database. The loci were mapped to 237 KEGG pathways. M; Metabolism, GIP; Genetic Information Processing, EIP; Environmental Information Processing, CP; Cellular Processes, OS; Organismal Systems.

In addition, to identify active biochemical pathways, we mapped the *H. tuberosus* loci onto the KEGG pathways using BLASTX and the KEGG Automatic Annotation Server [47], [48]. KO identifiers were assigned to 11,844 loci, using the KEGG orthology that contains 4,531 Enzyme Codes [46]. A number of KEGG pathways (237) were associated > 5 loci. The prevalent pathways represented were ‘Ribosome’ (408 loci), ‘Plant hormone signal transduction’ (365 loci), ‘Plant-pathogen interaction’ (365 loci), ‘Protein processing in endoplasmic reticulum’ (354 loci), ‘Spliceosome’ (329 loci), ‘Neurotrophin signaling pathway’ (285 loci), and ‘Starch and sucrose metabolism’ (276 loci) (Table S3). The number of sequences associated with subcategories in the top five KO categories are shown in Figure 4. Among the identified functional categories, ‘Signal transduction’ (1,252 loci), ‘Translation’ (1,029 loci), ‘Carbohydrate metabolism’ (1,023 loci), and ‘Folding, sorting and degradation’ (913 loci) were the most highly represented. These results showed that loci involved in processing of genetic information, pathogen resistance, and carbohydrate metabolism were active in *H. tuberosus* in the sampled tissues. The KEGG annotations provided valuable information for investigation of metabolic processes, functions and pathways involved in *H. tuberosus* metabolism.

### 3.5 Identification of Differentially Expressed Loci using RNA-Seq Data

**Figure 5:**
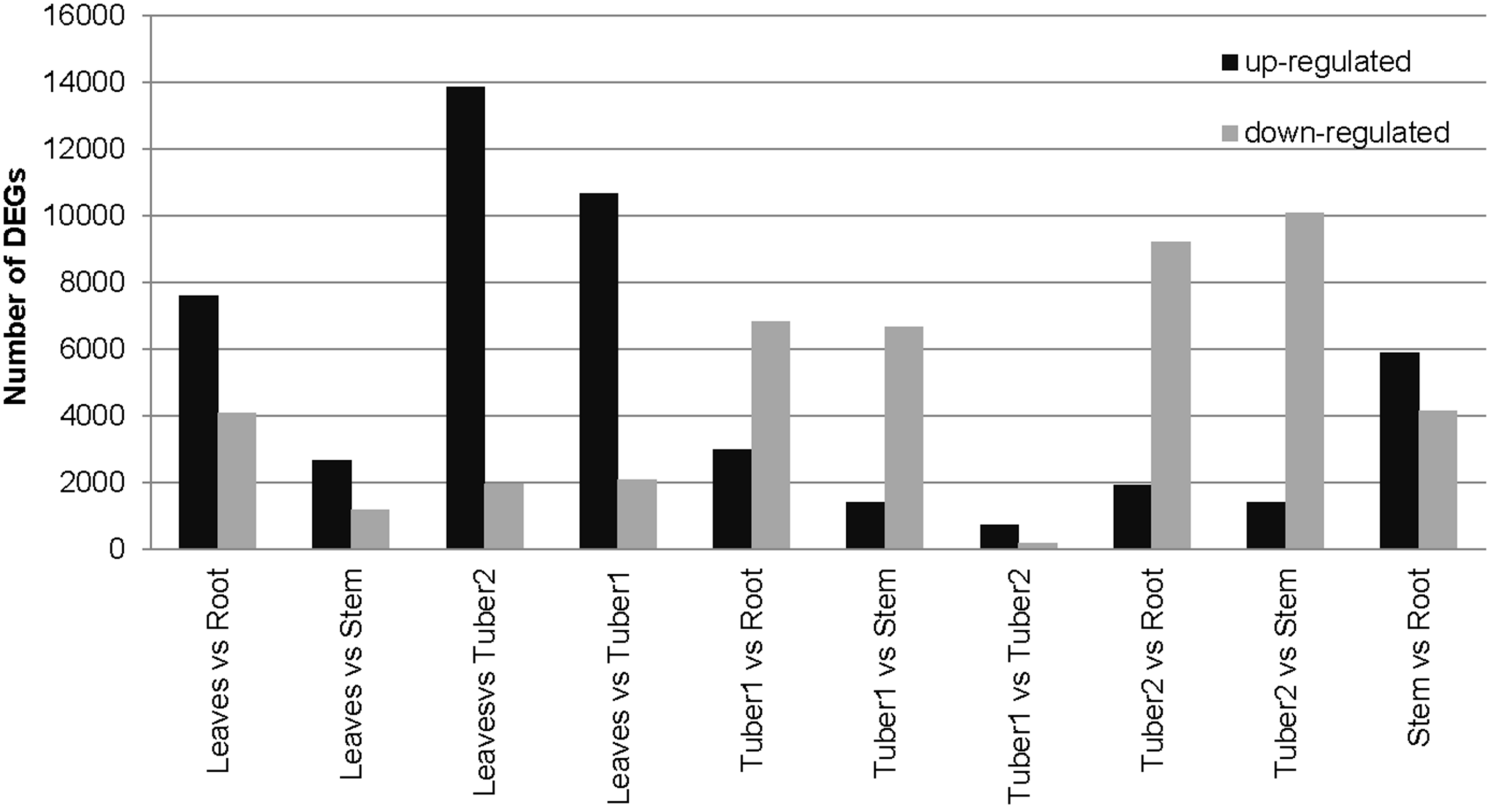
Loci differentially expressed between tissues in *H. tuberosus*. Loci were quantified and up- and down-regulated loci are shown as black and grey bars, respectively. Pairwise comparisons between tissues are shown.

RNA-Seq data were used for the identification of differentially expressed genes (DEGs) in different *H. tuberosus* tissues. More than 4.8 million raw reads were obtained from the libraries for each tissue (roots, stems, tuber1, tuber2, and leaves) (Table S1). To create a unified library, the reads were normalized by the total read count for gene expression in each tissue library (Figure S3). Next, Likelihood Ratio Tests were used to correct *p*-values, and libraries were median normalized. DEGs were identified using the following filters: adjusted *p*-value≤0.001, FDR ≤0.01, and log_2_ ratio at 2, ≤−2. Pairwise comparisons were performed between the five libraries. The average number of loci showing significant differences in expression between tissue pairs was 9,588 (range, 949–15,840) (Figure 5).

Comparison of differential expression between tissues showed that the largest expression difference occurred between leaves and tuber2 with 13,863 and 1,977 loci up- and down-regulated in leaves, respectively. The top four up-regulated loci in leaves were annotated as encoding pyridoxal-5′-phosphate-dependent enzyme family protein, acclimation of photosynthesis to environment (APE1) protein, single hybrid motif superfamily protein, and subunit NDH-M of NAD(P)H, suggesting an important role for these proteins in leaves. The most similar expression patterns were noted between tuber1 and tuber2 with only 949 differentially expressed loci identified (758 and 191 loci up- and down-regulated in tuber1, respectively) (Figure 5). The similarity in gene expression pattern between the two tuber tissues suggests that metabolic processes are similar at both stages of development stages. Differentially expressed loci were subjected to functional enrichment analysis using R tools. For pathway enrichment analysis, the specifically expressed loci were assigned to terms in the KEGG database and KEGG terms were identified that were significantly enriched compared to the underlying transcriptome. A hypergeometric test was applied and *p*-values were adjusted using the Bonferroni method [61], [62] to identify significantly enriched pathways. Functional loci involved in the ‘Photosynthesis and Photosynthesis – antenna proteins’ pathway were enriched in leaves compared to the other four tissues. Loci involved in the ‘alpha-Linolenic acid metabolism’ and ‘Plant hormone signal transduction’ pathways were enriched in stems, ‘Phenylalanine metabolism’ and ‘Amino sugar and nucleotide sugar metabolism’ pathways were enriched in roots, ‘Protein processing in endoplasmic reticulum’ and ‘Zeatin biosynthesis’ pathways were enriched in tuber1, and ‘Ribosome’ and ‘Flavone and flavonol biosynthesis’ pathways were enriched in tuber2.

Notably, α-linolenic acid metabolism-related loci were specific to the stem tissue. α-Linolenic acid is released from plant lipids in response to stress stimuli or biotic elicitation. In addition, α-linolenic acid initates a signal cascade that stimulates the production of secondary metabolites involved in plant defense. A previous study reported that the defense hormone methyl-jasmonate plays a role in the biosynthesis and accumulation of inulin in Jerusalem artichoke [63]. Secondary metabolites with medicinal uses are derived from phenylalanine and are synthesized mainly in the root [64]. In the current study, functional enrichment analysis demonstrated that loci involved in zeatin biosynthesis tuber development [65] were enriched in early stage tuber1, and flavonoid biosynthesis-related loci, which could enhance the efficiency of nutrient retrieval and transport [66], were enriched in later stage tuber2. Previous research showed that, potato tubers expressed genes involved in expressed genes of potato included starch biosynthesis genes and synthesis of storage proteins [59]. Similarly, our results also showed expression of loci related to biosynthesis and transport within tubers.

### 3.6 Validation of Expression of Tissue-Specific Candidate Loci by RT-PCR and qRT-PCR

Quantitative reverse-transcription-PCR (qRT-PCR) was performed to validate DEGs from different *H. tuberosus* tissues, and to evaluate the reliability of the *H. tuberosus* transcriptome assembly. Candidate tissue-specific loci were chosen with read count values > 200 in one tissue and < 50 in other tissues (Table S4). Twenty tissue-specific candidates were selected from the five tissues. Primer sets were designed to verify tissue-specific expression (Table S5) and were used for RT-PCR validation (Figure S4). Quantification of tissue-specific loci was conducted using qRT-PCR with two tissue-specific loci for each tissue.

**Figure 6:**
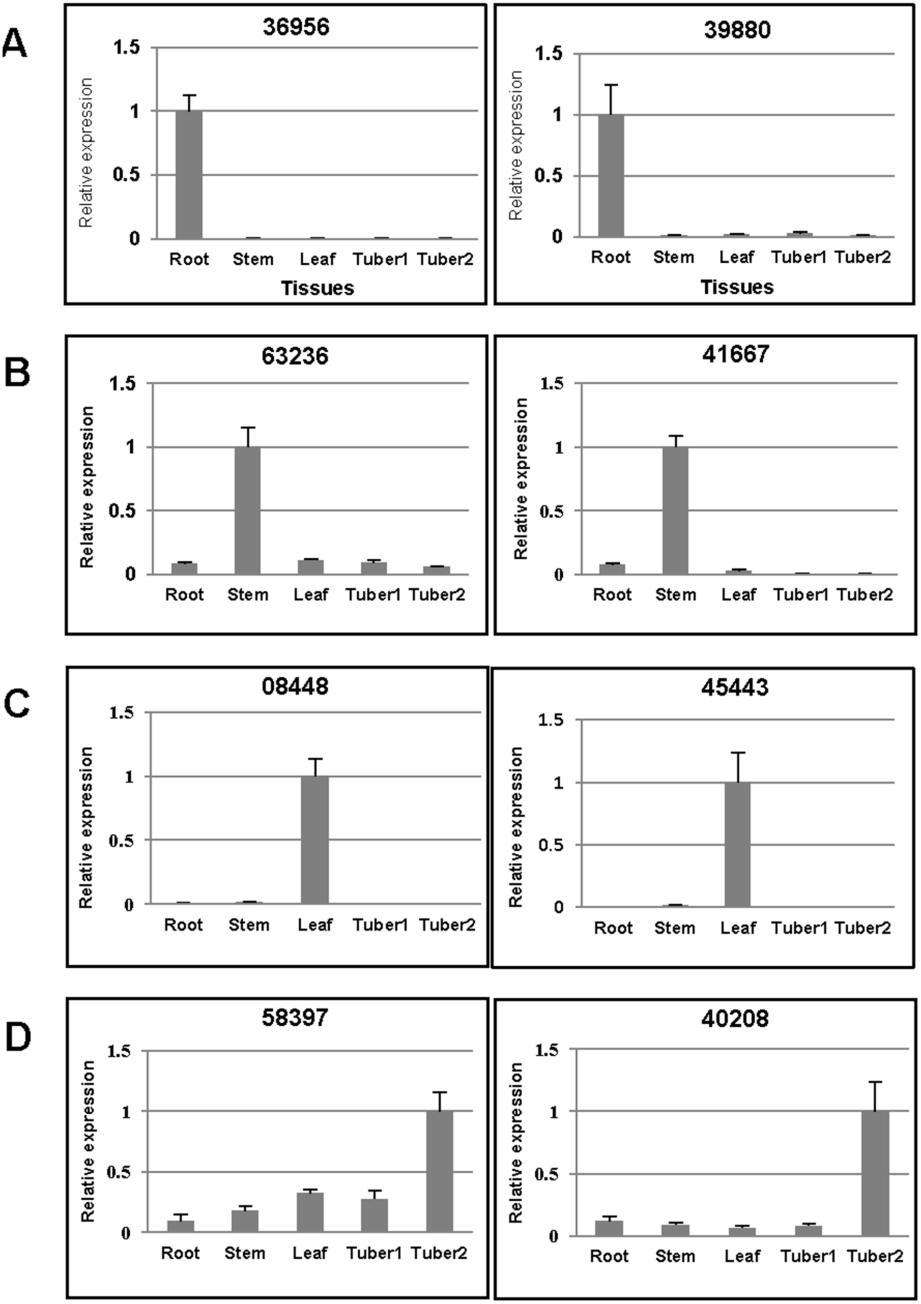
qRT-PCR validation of loci expressed specially in five *H. tuberosus* tissues. The qRT-PCR results of root-specific (**A**), stem-specific (**B**), leaf-specific (**C**), and tuber-specific (**D**) candidate loci are shown.

Locus 36956 (similar to Arabidopsis 1-AMINOCYCLOPROPANE-1-CARBOXYLATE OXIDASE (AT2G19590), which is involved in cell wall macromolecule metabolic processes), and locus 39880 (similar to AT4G12520, which is annotated as ‘bifunctional inhibitor/lipid-transfer protein/seed storage 2S albumin superfamily protein) were confirmed as uniquely expressed in root tissue (Figure 6A). Similarly, locus 63236 (similar to CYSTEINE PROTEINASES SUPERFAMILY PROTEIN (AT5G50260)), and locus 41667 (similar to HPT PHOSPHOTRANSMITTER 4 (AT3G16360)), were highly expressed in stem (Figure 6B). Locus 08448 (similar to MLP-LIKE PROTEIN 28 (AT1G70830)), and locus 45443 (similar to PLANT PROTEIN OF UNKNOWN FUNCTION (AT3G02645)) were confirmed to be predominently expressed in leaf tissue (Figure 6C). Locus 58397 (similar to INTEGRASE-TYPE DNA-BINDING SUPERFAMILY PROTEIN (AT5G52020)), and locus 40208 (similar to an F-box and associated interaction domains-containing protein (AT4G12560)) were highly expressed in tuber tissues, in either a stage-specific or non-stage-specific pattern (Figure 6D). The Arabidopsis genes similar to each annotated locus are shown in Table S5.

### 3.7 SSR Markers in the *H. tuberosus* Transcriptome

*H. Tuberosus* sequences (66,322 loci) were examined for SSRs. A total number of 10,778 SSRs were identified from 8,746 unique loci. Of these, 1,604 loci contained more than one of SSR motif (Table S6). The SSR frequency in the *H. tuberosus* transcriptome was 16.25% and the average distance between SSRs was 4.68 kb. Di-nucleotide repeats constituted the most abundant class, followed by tri-nucleotide repeats (Figure S5A, Table S7). In addition, among the specific repeat motifs, di- and tri-nucleotide repeats were the most common, with AG/CT motifs accounting for 41.31% of the di-nucleotide repeats, fllowed by ATC/ATG (11.1%), ACC/GGT (9.41%), and AAG/CTT (8.25%) (Figure S5B). SSRs are thought to affect chromatin organization, gene regulation, recombination, DNA replication, the cell cycle, and mismatch repair [67]. In addition, SSR markers are invaluable for genetic diversity analysis [68].

Our transcriptome survey revealed that di-nucleotide repeats (37.53%) are more abundant in Jerusalem artichoke than are tri- (31.13%), mono- (28.38%), tetra- (1.9%), penta- (0.55%) and hexanucleotide repeats (0.51%). These microsatellite characteristics concur with those in the transcriptomes of several other plants [69]–[71]. Our SSR data therefore represent an important resource for the development of molecular markers for research and molecular breeding of Jerusalem artichoke.

### 3.8 Loci from the *H. tuberosus* Transcriptome Involved in the Inulin Biosynthesis Pathway

**Figure 7:**
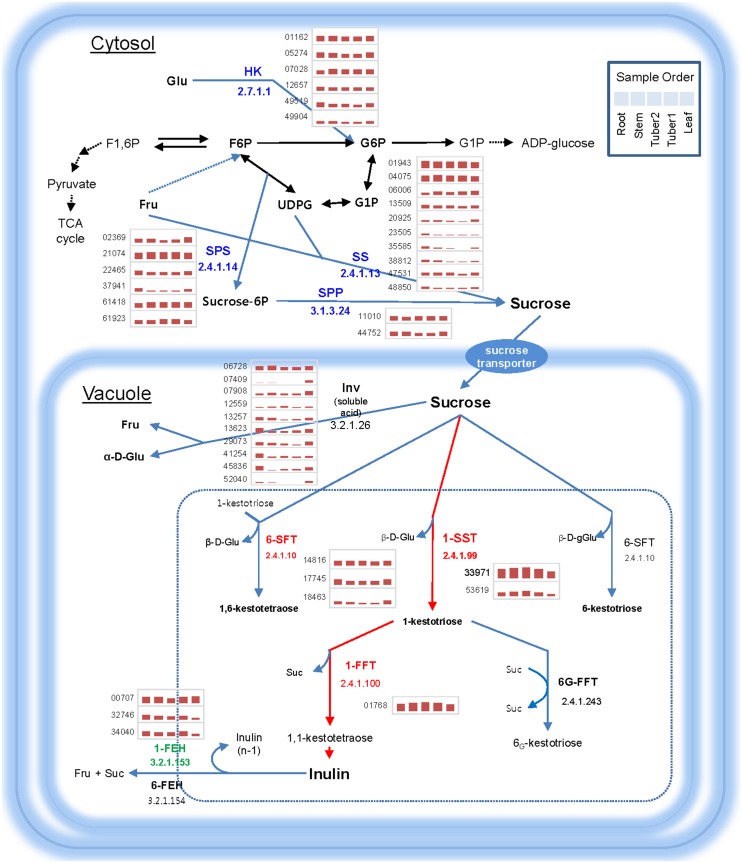
Schematic representation of the inulin biosynthesis pathway in the vacuole. Inulin biosynthesis enzymes present in the vacuole are marked in red. Green indicates enzymes related to inulin degradation. Blue indicates enzymes related to sucrose biosynthesis. Read counts of unigenes representing enzymes were subjected to expression analysis and the results are shown as red bars (log_2_). 1-SST: 1-sucrose: sucrose fructosyltransferase, 6-SFT: sucrose:sucrose fructosyltransferase, 1-FFT: 1,2-β–fructan 1F-fructosyltransferase, 6G-FFT: Fructan:fructan 6G-fructosyltransferase, FEH: fructan exohydrolase, HK: Hexokinase, SS: Sucrose synthase, SPS: Sucrose-phosphate-synthase, SPP: Sucrose-phosphate-phosphohydrolase, Suc: Sucrose, Fru: Fructose, Glu: Glucose, Inv: Invertase.

Inulin has phamarceutical applications in treating diabetes and obesity. In *H. tuberosus*, inulin mainly accumulates in tuber tissue, and it was therefore of interest to identify the genes responsible for biosynthesis and vacuolar storage of inulins in tubers. We used our RNA-Seq data to conduct expression profiling of loci related to carbohydrate metabolism (Figure 7). Cytosolic sucrose is the only substrate for inulin biosynthesis. Two major enzymes, fructan 1, 2-beta-fructan 1-fructosyltransferase (1-FFT) and sucrose:sucrose 1F-beta-D-fructosyltransferase (1-SST), function in transport of sucrose [72]. The proteins encoded by the loci involved in sucrose biosynthesis are likely to be present mainly in the cytosol, whereas the proteins involved in fructose chain formation are likely be present in the vacuole. We analyzed the expression of loci encoding major carbohydrate metabolic enzymes in different tissues to understand the inulin biosynthesis pathway in *H. tuberosus*. The four key enzymes involved in sucrose biosynthesis, hexokinase (6 loci), sucrose phosphate synthase (6 loci), sucrose synthase (10 loci) and sucrose phosphate phosphatase (2 loci), were expressed in most tissues; however, some of the loci showed tissue-specificity and had very low expression levels (Figure 7). Two essential enzymes in inulin biosynthesis, 1-FFT and 1-SST, were more strongly expressed in tuber1 and tuber2 tissues than in other tissues. Locus 33971, annotated as 1-SST, showed 7.6-fold and 59-fold higher expression in tuber tissue than in root and leaf tissues, respectively. Another key enzyme, 1-FFT (locus 01768), also showed tuber tissue-specificity with high expression levels. Locus 01768 was expressed more than 18.6-fold and 9-fold higher in tuber tissue than in leaf and root tissues, respectively. Interestingly, the fructan 1-exohydrolase (1-FEH) enzyme involved in inulin degradation was not highly expressed; rather, gene expression was lower in tuber tissue than in other tissues (Table 3). In summary, we identified loci corresponding to 1-FFT and 1-SST, which are two key enzymes involved in inulin biosynthesis, and demonstrated that these unigenes were more highly expressed in tuber than in other tissues. These results are consistent with inulin biosynthesis occurring mainly in tuber tissue.

**Table 3:** Identification of genes involved in inulin biosynthesis in *H. tuberosus*.

Enzyme	EC number	Locus ID	Read Count (log_2_)				
			Root	Stem	Tuber2	Tuber1	Leaf
Hexokinase	2.7.1.1	01162	10.41	9.97	8.38	8.67	9.46
		05274	10.28	8.45	7.84	8.06	9.79
		07028	7.97	10.24	8.56	8.42	9.00
		12657	7.77	6.55	5.70	6.29	6.58
		49519	8.80	6.30	6.29	5.25	7.04
		49904	5.17	4.46	3.58	4.17	5.25
Sucrose Phosphate Synthase	2.4.1.14	02369	7.13	7.71	5.17	5.73	10.08
		21074	11.07	12.00	12.44	12.39	11.17
		22465	8.67	6.25	6.83	6.55	6.98
		37941	6.55	2.00	2.00	3.00	4.70
		61418	8.95	10.03	10.17	10.28	9.56
		61923	7.17	8.07	8.35	8.28	7.23
Sucrose Synthase	2.4.1.13	01943	14.89	13.14	13.67	14.21	11.91
		04075	11.89	13.29	12.27	11.93	11.50
		06006	4.75	7.75	5.70	7.13	8.16
		13509	7.55	6.04	6.04	4.86	5.64
		20925	3.81	4.00	3.00	1.00	4.58
		23505	3.70	1.00	1.00	1.00	1.00
		35585	4.58	3.00	1.00	0.00	2.58
		38812	4.39	3.58	1.00	1.58	2.58
		47531	6.83	6.36	5.64	3.70	6.78
		48850	4.46	2.58	2.32	2.32	3.58
Sucrose Phosphate Phosphatase	3.1.3.24	11010	8.70	8.03	8.43	8.26	8.22
		44752	8.61	8.62	6.48	6.02	8.83
Fructan: fructan 1, 2-beta-fructan 1-fructosyltransferase	2.4.1.100	01768	11.86	13.88	15.01	13.76	10.80
Sucrose: sucrose 1F-beta-D-fructosyltransferase	2.4.1.99	33971	14.51	16.13	17.44	15.35	11.56
		53619	5.64	7.25	8.46	6.39	3.32
Sucrose 6-fructosyltransferase	2.4.1.10	14816	7.55	7.98	6.94	6.39	7.43
		17745	8.25	6.73	5.81	6.00	8.81
		18463	4.91	4.32	2.58	3.00	5.78
Fructan 1-exohydrolase Iia	3.2.1.153	00707	11.55	10.70	8.69	11.65	11.90
		32746	8.73	7.54	5.81	7.60	3.81
		34040	9.18	7.92	8.08	8.48	5.52
Soluble acid Invertase	3.2.1.26	06728	10.38	10.95	7.92	7.85	9.66
		07409	1.58	1.58	0.00	0.00	5.73
		07908	5.52	6.73	5.09	4.70	10.17
		12559	2.00	3.32	4.46	2.58	3.70
		13257	7.81	6.21	3.00	3.81	6.81
		13623	10.65	5.32	5.00	5.73	8.23
		29073	7.63	6.46	5.09	3.00	8.12
		41254	9.99	4.32	4.70	3.70	4.70
		45836	9.69	1.58	3.32	3.32	8.04
		52040	2.00	1.00	0.00	0.00	4.95

Expression values were log_2_ transformed and are provided as a normalized read number. EC, Enzyme Codes.

## Conclusions

We took advantage of RNA-Seq technology from the Illumina platform to investigate metabolic pathways and tissue-specific gene expression in a non-model plant species. Our transcriptome analysis used raw data at an unprecedented depth (20.6 Gbp) and produced a total of 66,322 assembled loci using *de* *novo* assemblers. Of these loci, 87.65% were novel sequences not present in the most recently released *H. tuberosus* EST database. We mapped 11,844 loci onto 237 KEGG pathways, including ‘Carbohydrate metabolism’ and ‘Signal transduction and Translation pathways’. We further found 43 loci that functioned in sucrose and inulin metabolism. We performed RT-PCR with 20 tissue-specific candidate loci, and most tissue-specific candidate loci were expressed mainly in specific *H. tuberosus* tissues. In addition, qRT-PCR results confirmed the reliability of the *H. tuberosus* transcriptome assembly and tissue-specificity of expressed loci. SSR markers were identified, and these could provide primary information for analysis of polymorphisms within Jerusalem artichoke populations. The assembled transcriptome sequences and additional data make a substantial contribution to the existing genomic resources for *H. tuberosus* and will serve to enable functional genomics research in *H. tuberosus*.

## Supporting Information

Figure S1 **Length distributions of transcripts assembled with Trinity and Velvet-Oases algorithms.**
(TIF)

Figure S2 **Venn diagrams comparing** ***de*** ***novo*** **assembled sequences and available ESTs of** ***H. annuus.*** **and** ***H. tuberosus.***
(TIF)

Figure S3 **Hierarchical cluster analysis of expressed loci in** ***H. tuberosus*** **tissues.**
(TIF)

Figure S4 **RT-PCR analysis of loci expresses in specific tissues.**
(TIF)

Figure S5 **Summary of simple sequence repeats identified in the** ***H. tuberosus*** **transcriptome.**
(TIF)

Table S1 **Summary of** ***H. tuberosus*** **transcriptome sequencing.**
(TIF)

Table S2 **Sequence annotations of the** ***H. tuberosus*** **transcriptome against the Phytozome database and locus expression levels (Read count).**
(XLS)

Table S3 **Summary of loci involved in KEGG pathways and KO categories.**
(XLS)

Table S4 **Summary of pairwise comparisons of loci expressed in specific** ***H. tuberosus*** **tissues.**
(TIF)

Table S5 ***H. tuberosus*** **tissue-specific locus candidates and their primers.**
(TIF)

Table S6 **SSR search statistics.**
(TIF)

Table S7 **Distribution of different repeat type classes in the *H. tuberosus* transcriptome.**
(TIF)
