# Supplementary figures and images for: RNA-Seq Analysis and De Novo Transcriptome Assembly of Jerusalem Artichoke (Helianthus tuberosus Linne)
Source: PLoS One. 2014 Nov 6;9(11):e111982. doi: 10.1371/journal.pone.0111982 (PMC4222968; doi:10.1371/journal.pone.0111982)

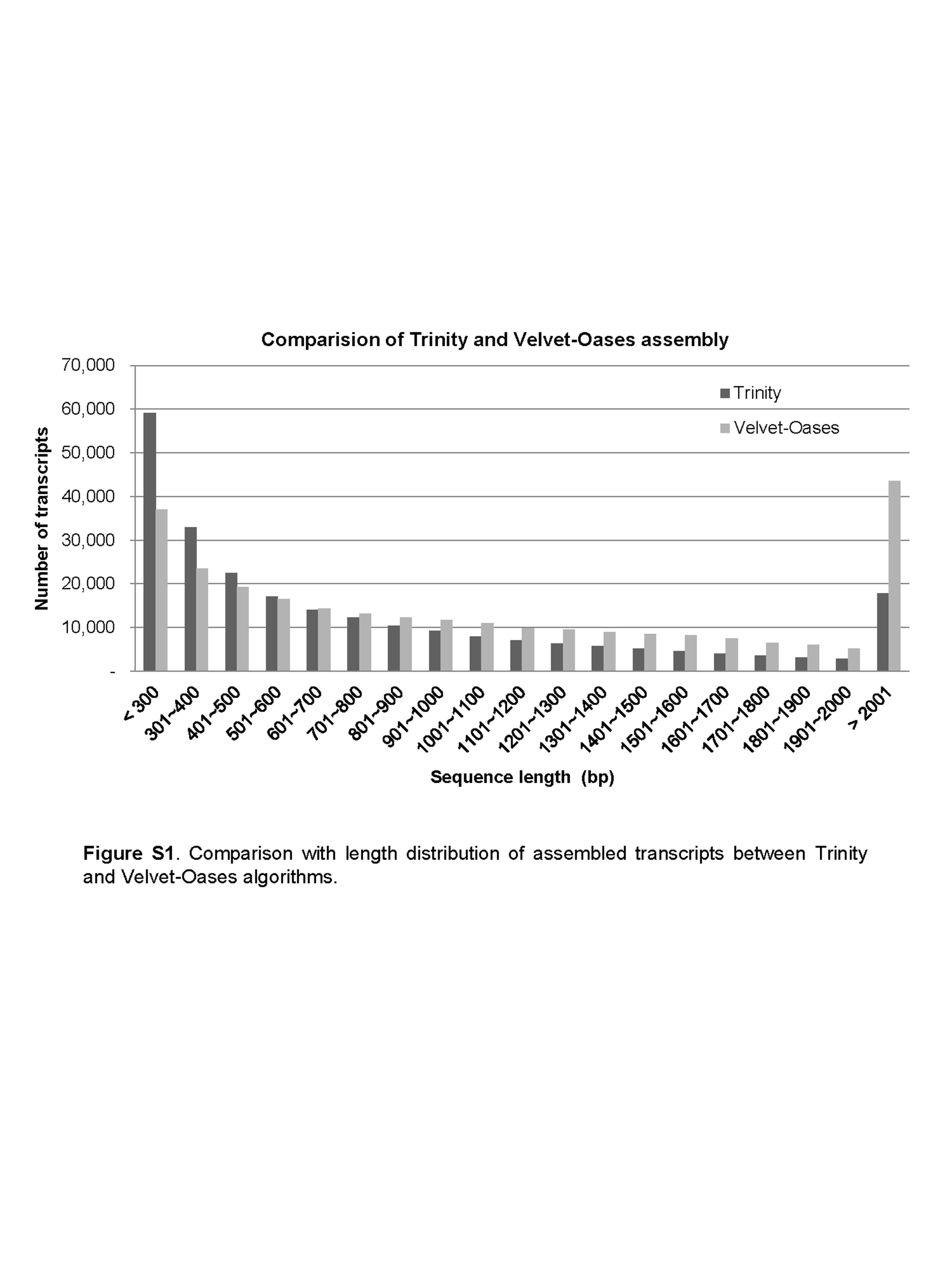

Supplement: Figure S1 — Length distributions of transcripts assembled with Trinity and Velvet-Oases algorithms. (TIF) [file pone.0111982.s001.tif]

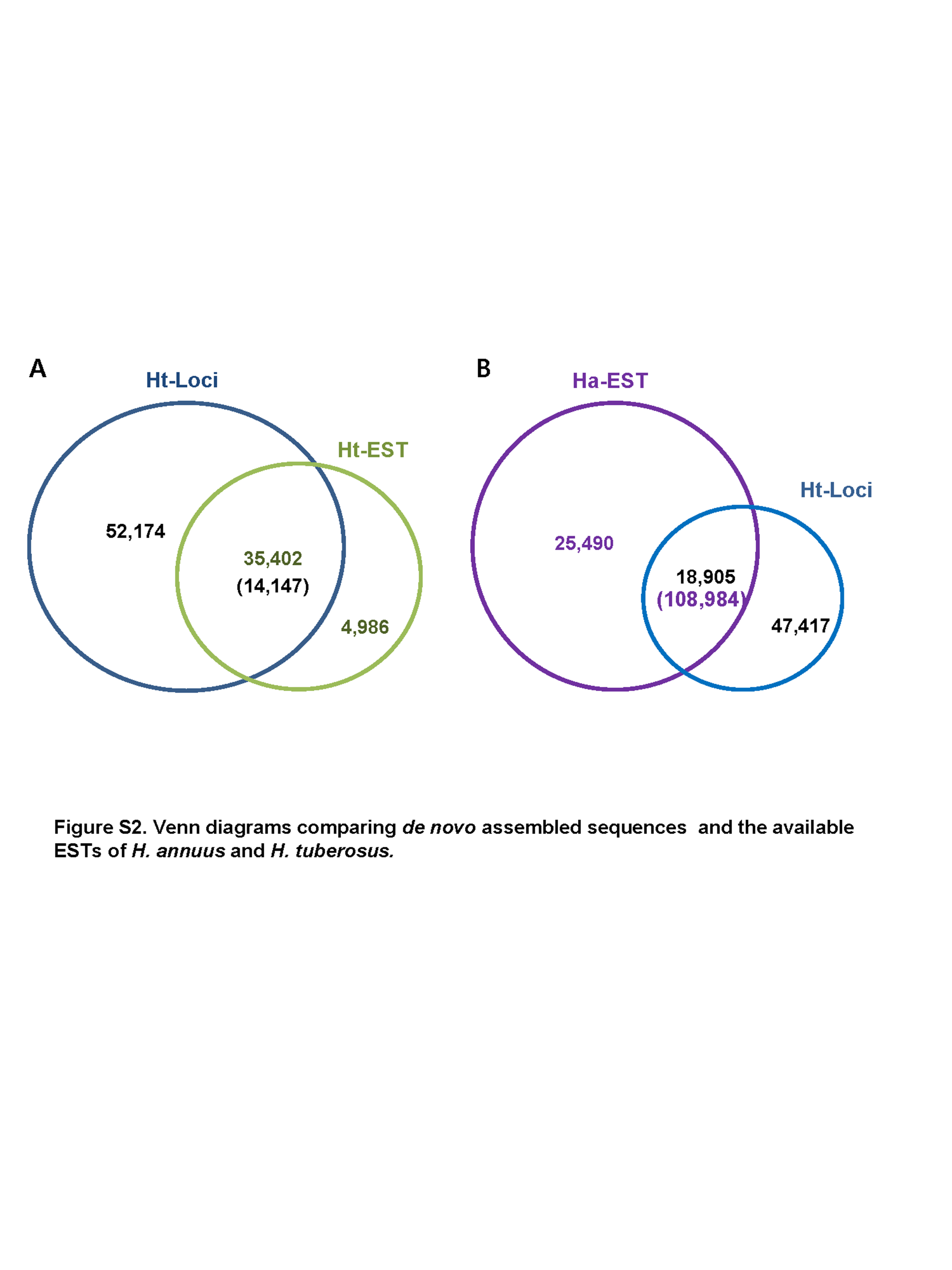

Supplement: Figure S2 — Venn diagrams comparing de novo assembled sequences and available ESTs of H. annuus. and H. tuberosus. (TIF) [file pone.0111982.s002.tif]

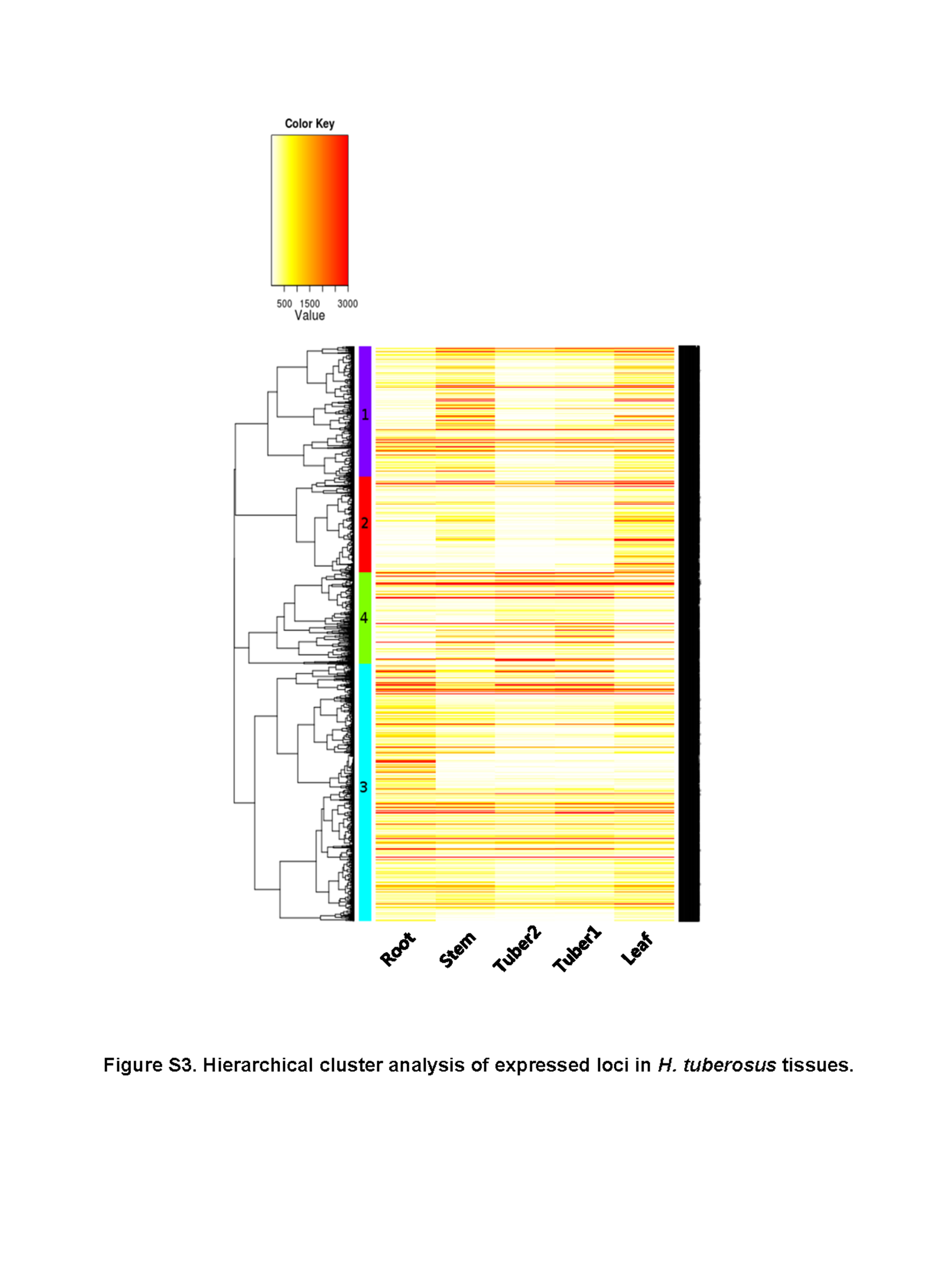

Supplement: Figure S3 — Hierarchical cluster analysis of expressed loci in H. tuberosus tissues. (TIF) [file pone.0111982.s003.tif]

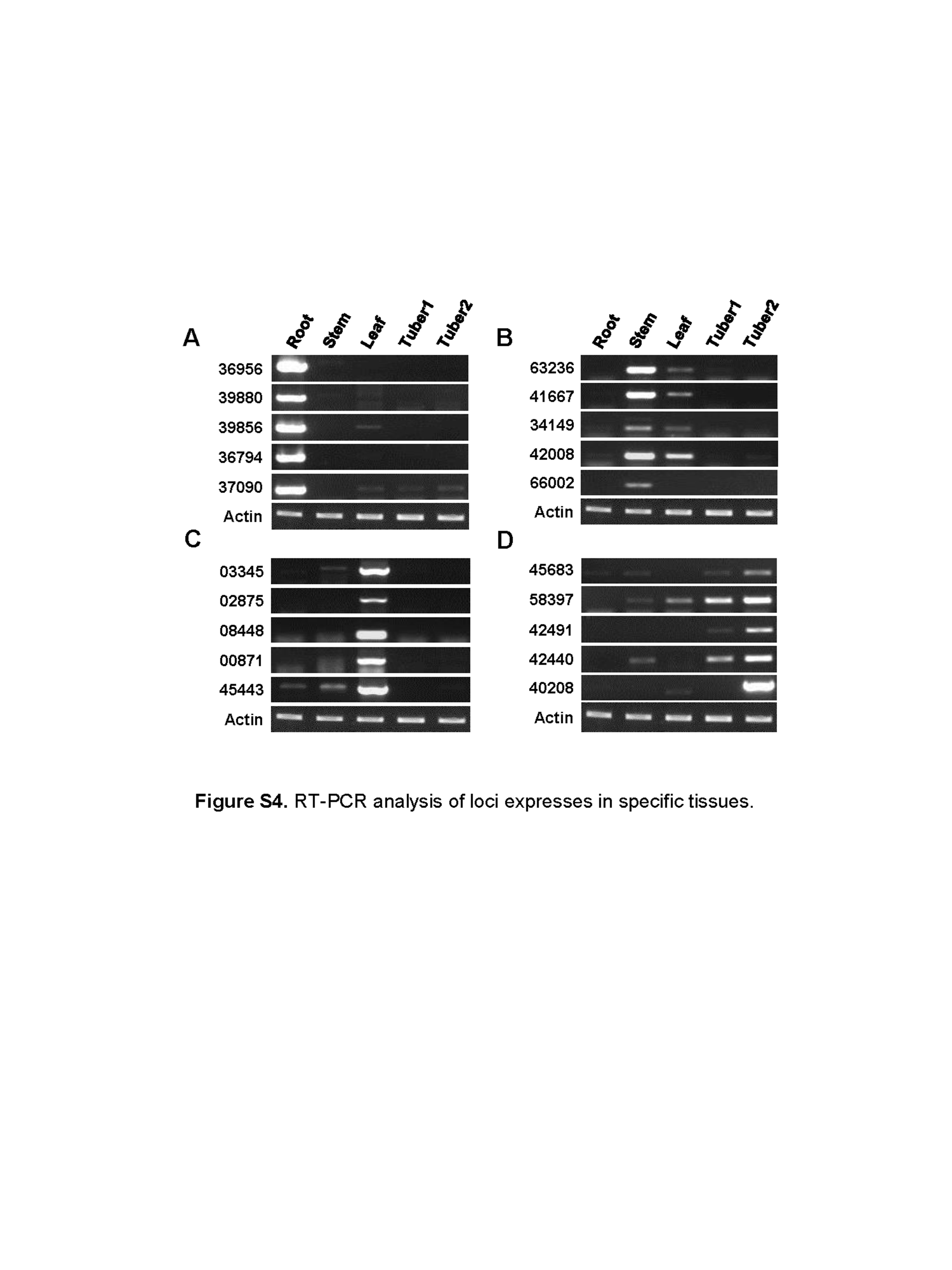

Supplement: Figure S4 — RT-PCR analysis of loci expresses in specific tissues. (TIF) [file pone.0111982.s004.tif]

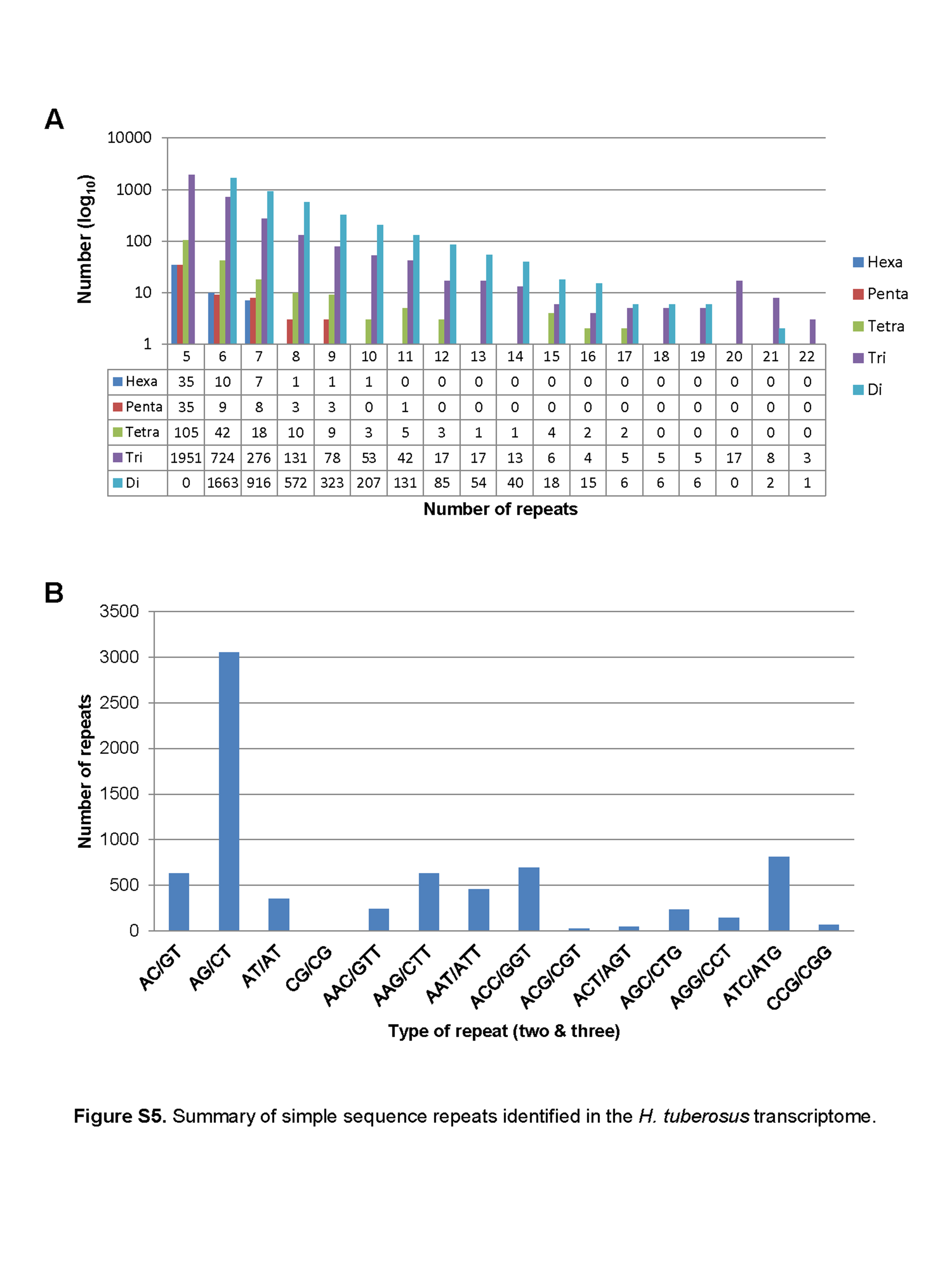

Supplement: Figure S5 — Summary of simple sequence repeats identified in the H. tuberosus transcriptome. (TIF) [file pone.0111982.s005.tif]

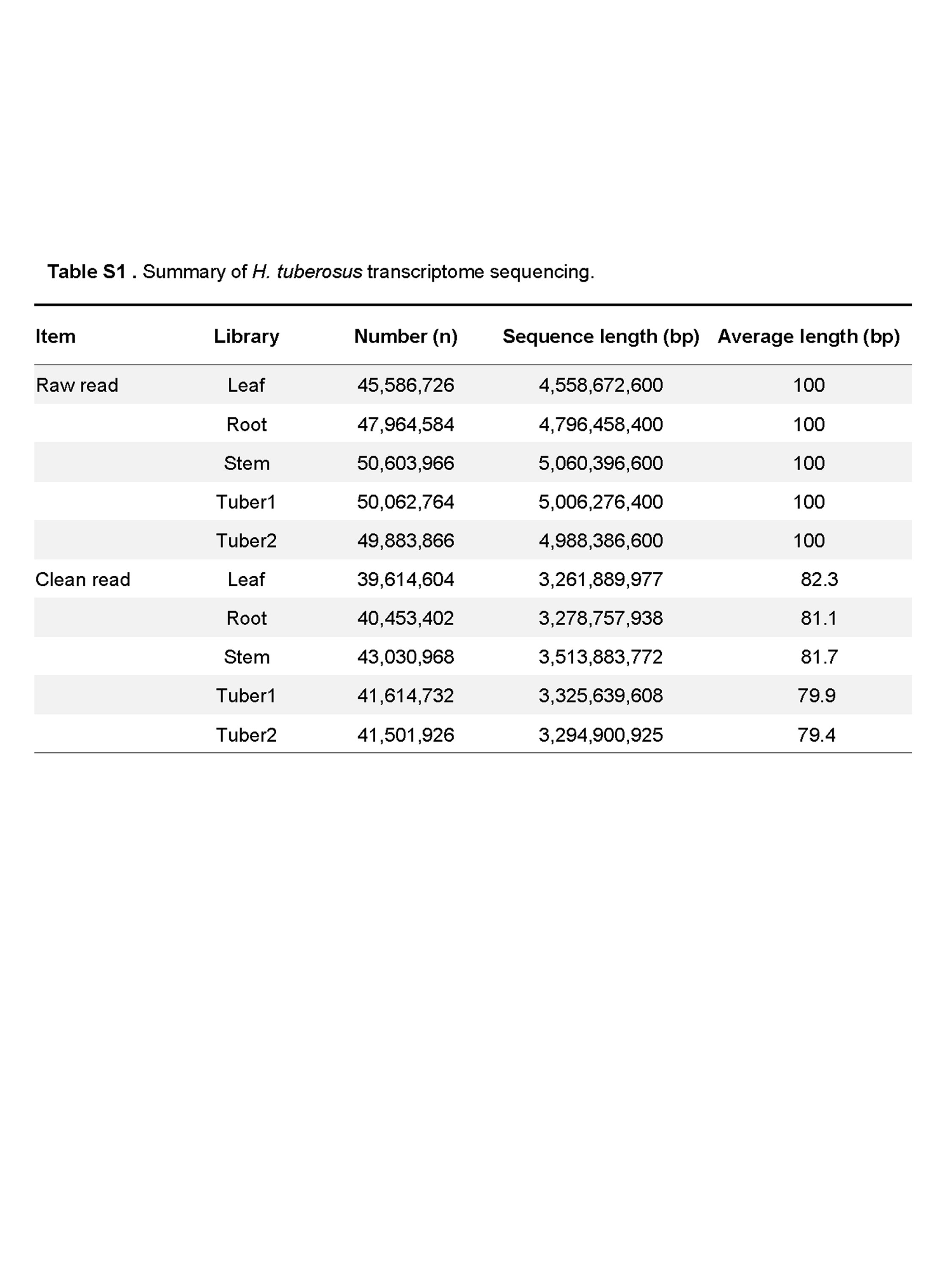

Supplement: Table S1 — Summary of H. tuberosus transcriptome sequencing. (TIF) [file pone.0111982.s006.tif]

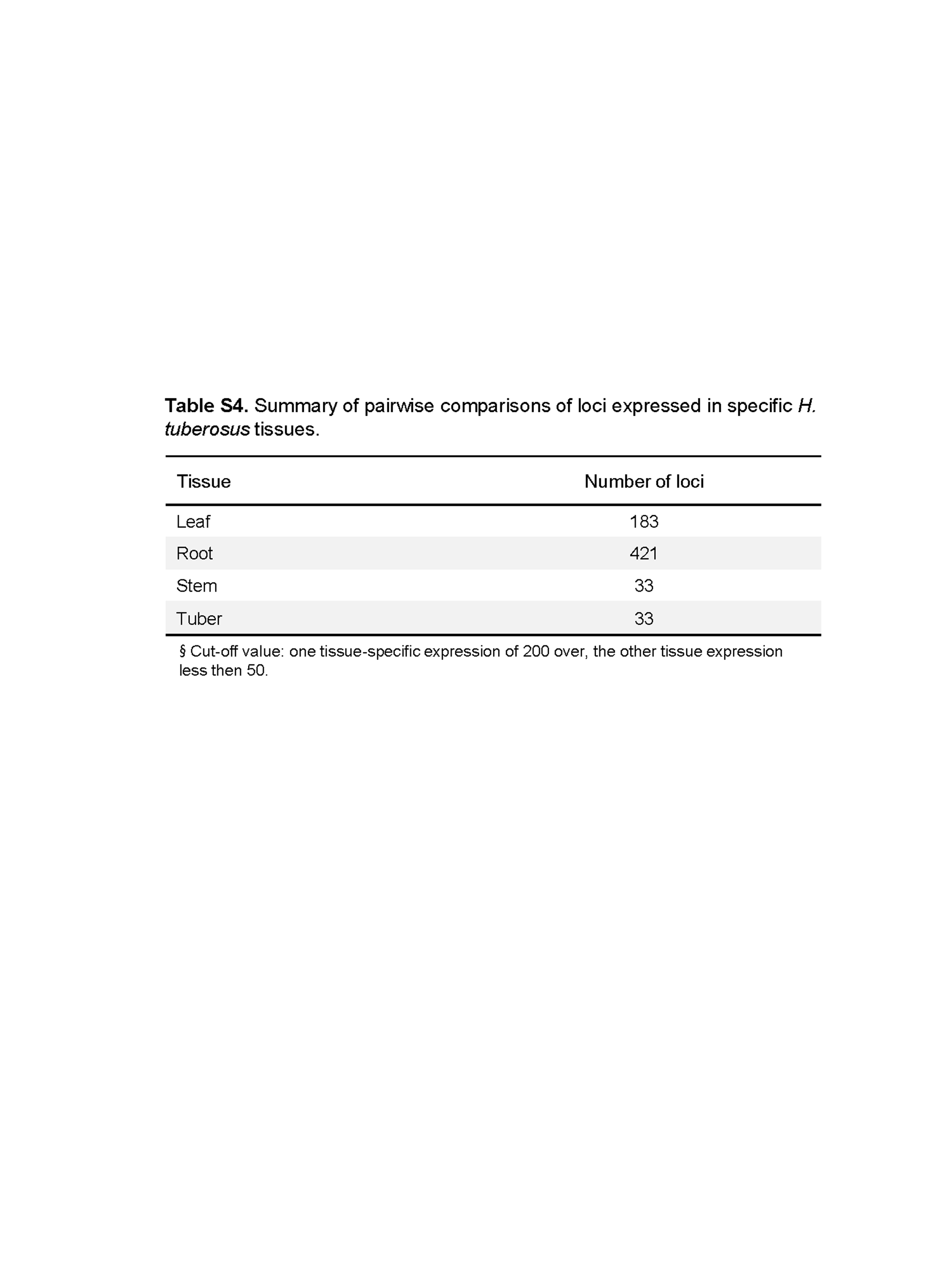

Supplement: Table S4 — Summary of pairwise comparisons of loci expressed in specific H. tuberosus tissues. (TIF) [file pone.0111982.s009.tif]

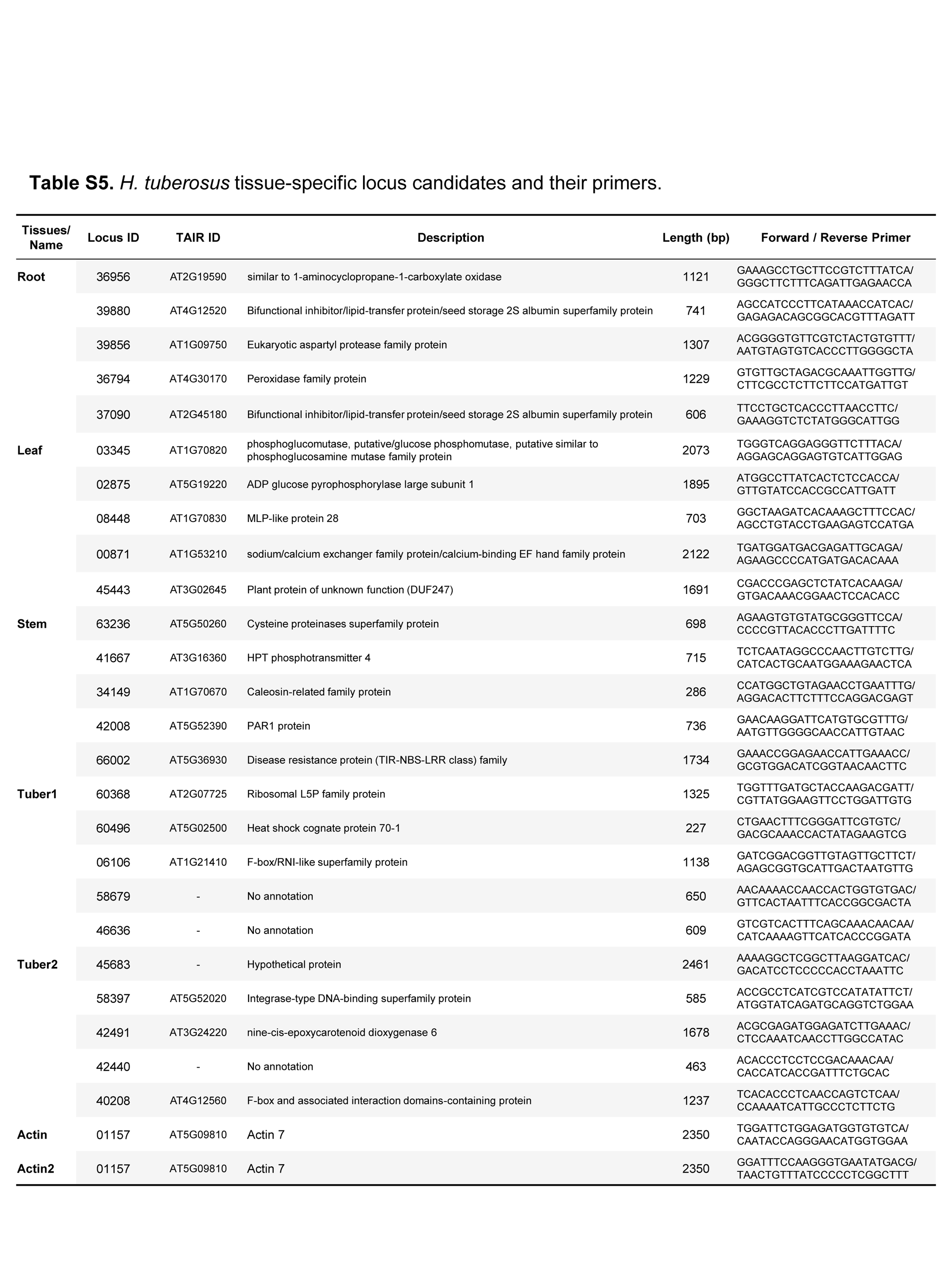

Supplement: Table S5 — H. tuberosus tissue-specific locus candidates and their primers. (TIF) [file pone.0111982.s010.tif]

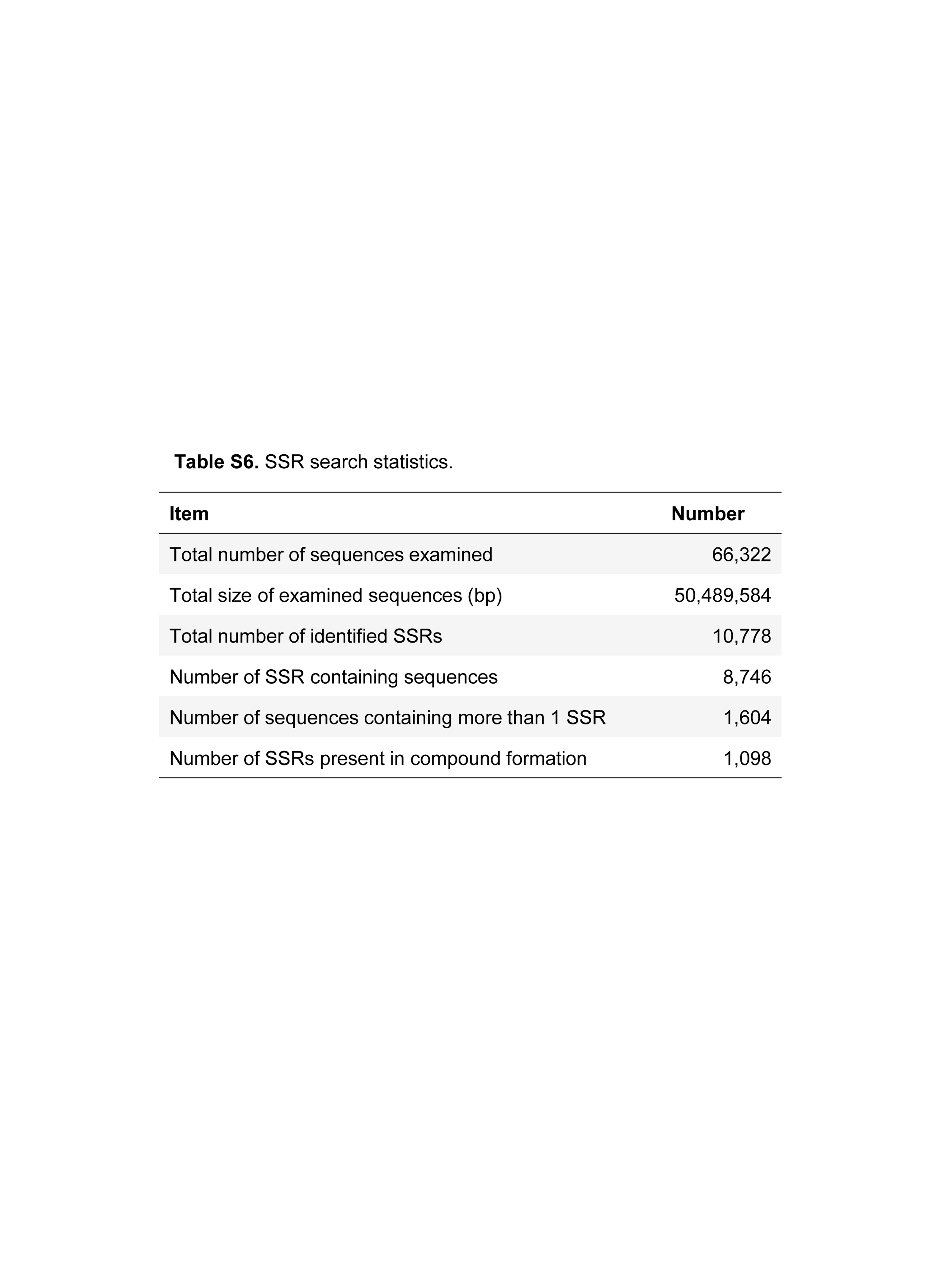

Supplement: Table S6 — SSR search statistics. (TIF) [file pone.0111982.s011.tif]

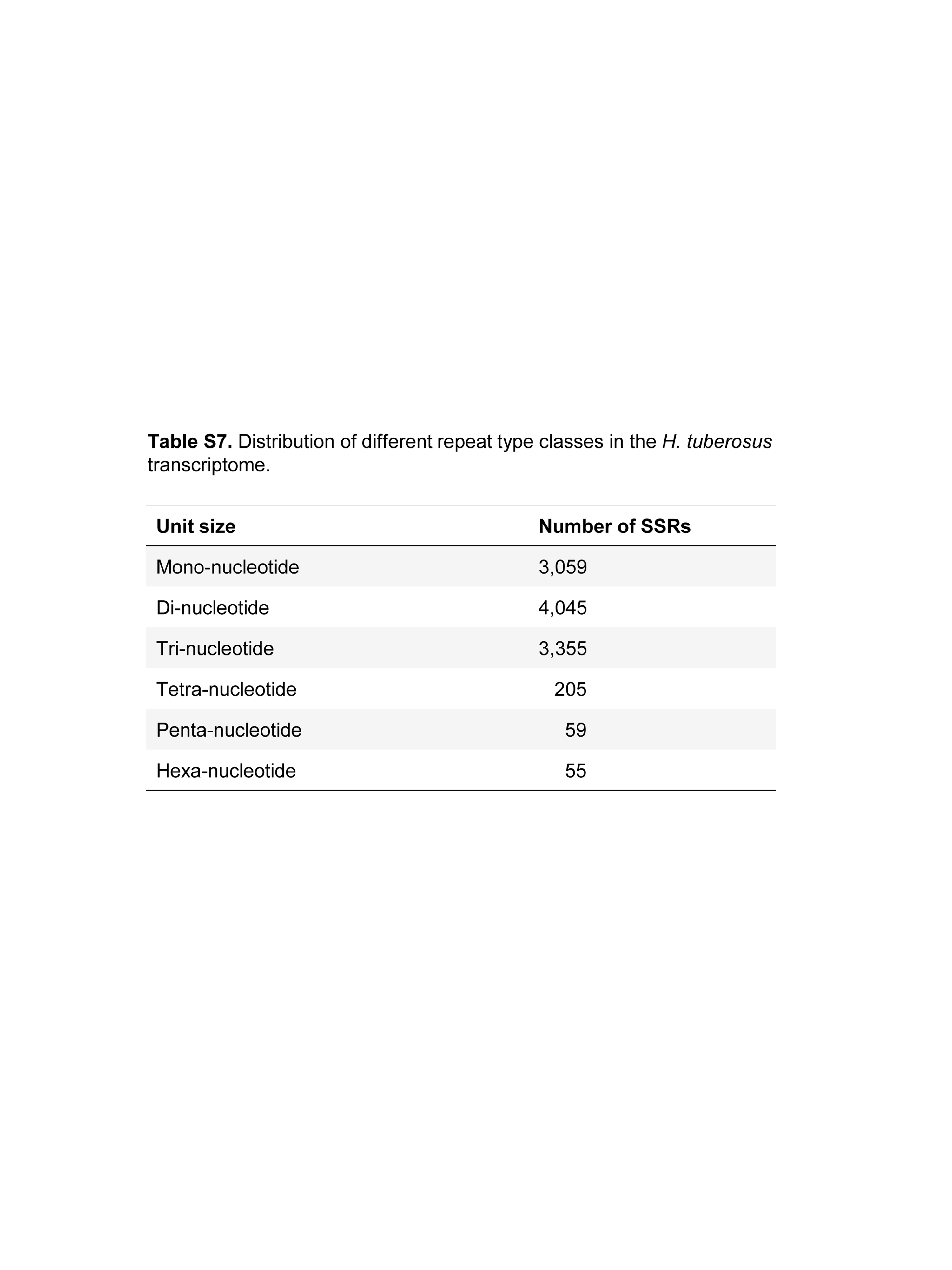

Supplement: Table S7 — Distribution of different repeat type classes in the H. tuberosus transcriptome. (TIF) [file pone.0111982.s012.tif]
